# Dosimetric benefits of adaptive radiation therapy for patients with stage III non-small cell lung cancer

**DOI:** 10.1186/s13014-023-02222-7

**Published:** 2023-02-22

**Authors:** Lea Hoppen, Gustavo R. Sarria, Chung S. Kwok, Judit Boda-Heggemann, Daniel Buergy, Michael Ehmann, Frank A. Giordano, Jens Fleckenstein

**Affiliations:** 1 Department of Radiation Oncology, University Medical Center Mannheim, University of Heidelberg, Theodor-Kutzer-Ufer 1-3, 68167 Mannheim, Germany; 2 Department of Radiation Oncology, University Hospital Bonn, University of Bonn, Bonn, Germany

**Keywords:** Adaptive radiation therapy, stage III NSCLC, Isotoxic dose-escalation, Isoeffective organ at risk sparing

## Abstract

**Background:**

Daily adaptive radiation therapy (ART) of patients with non-small cell lung cancer (NSCLC) lowers organs at risk exposure while maintaining the planning target volume (PTV) coverage. Thus, ART allows an isotoxic approach with increased doses to the PTV that could improve local tumor control. Herein we evaluate daily online ART strategies regarding their impact on relevant dose-volume metrics.

**Methods:**

Daily cone-beam CTs (1 × n = 28, 1 × n = 29, 11 × n = 30) of 13 stage III NSCLC patients were converted into synthetic CTs (sCTs). Treatment plans (TPs) were created retrospectively on the first-fraction sCTs (sCT_1_) and subsequently transferred unaltered to the sCTs of the remaining fractions of each patient (sCT_2−n_) (IGRT scenario). Two additional TPs were generated on sCT_2−n_: one minimizing the lung-dose while preserving the D_95%_(PTV) (isoeffective scenario), the other escalating the D_95%_(PTV) with a constant V_20Gy_(lung_ipsilateral_) (isotoxic scenario).

**Results:**

Compared to the original TPs predicted dose, the median D_95%_(PTV) in the IGRT scenario decreased by 1.6 Gy ± 4.2 Gy while the V_20Gy_(lung_ipsilateral_) increased in median by 1.1% ± 4.4%. The isoeffective scenario preserved the PTV coverage and reduced the median V_20Gy_(lung_ipsilateral_) by 3.1% ± 3.6%. Furthermore, the median V_5%_(heart) decreased by 2.9% ± 6.4%. With an isotoxic prescription, a median dose-escalation to the gross target volume of 10.0 Gy ± 8.1 Gy without increasing the V_20Gy_(lung_ipsilateral_) and V_5%_(heart) was feasible.

**Conclusions:**

We demonstrated that even without reducing safety margins, ART can reduce lung-doses, while still reaching adequate target coverage or escalate target doses without increasing ipsilateral lung exposure. Clinical benefits by means of toxicity and local control of both strategies should be evaluated in prospective clinical trials.

## Background

The aim of radiation therapy (RT) is to achieve a high tumor control probability (TCP) with the lowest possible normal tissue complication probability (NTCP) [1]. For patients with locally advanced non-small cell lung cancer (NSCLC) the dose limiting organs at risk (OAR) are usually lungs, heart and esophagus, which tend to develop pneumonitis [2, 3], cardiovascular toxicity [4–7], or acute esophagitis [8, 9] due to high radiation doses, respectively. Most NSCLC radiation therapies are performed with 60–70 Gy total dose in 1.8–2 Gy-fractions [5, 10, 11]. Survival rates of patients with locally advanced (stage III) NSCLC have improved considerably during the last years. Modern concurrent chemoradiotherapy (cCRT) results in 5-year overall survival (OS) rates of 32–33% [12, 13]; results can be further improved in patients who are eligible for immunotherapy (absence of toxic events ≥ grade 2) with durvalumab as a consolidation therapy resulting in 5-year OS rates of approximately 43% [13]. Despite these improvements, only one third of patients treated with cCRT or immunotherapy survives or remains free from progression five years after treatment [13].

Currently, treatment plans are typically generated on a pre-treatment computed tomography (CT) scan and delivered in all treatment fractions by means of daily image guided radiation therapy (IGRT), sometimes in combination with an active motion management concept. Intrafractional motion management is mostly realized by either an internal target volume (ITV) margin, beam tracking or gating concepts [14]. The latter two reduce the planning target volume (PTV) size and aim at a quasi-static situation. However, all these workflows ensure adequate patient positioning but neglect interfractional morphologic changes in the patient’s anatomy which may lead to discrepancies between the delivered and planned dose [1, 15, 16]. This is of particular relevance for NSCLC since these tumors are generally early responders to radiation [16, 17]. On average the gross target volume (GTV) of NSCLC patients shrinks 0.6–2.4% per day during RT [3].

In online adaptive radiation therapy (ART), treatment plans are adjusted to a possibly changed patient anatomy [1, 15, 16] on daily pre-treatment imaging. This is mostly performed with cone-beam computed tomography (CBCT)-based approaches. Compared to fan-beam CTs, CBTCs present limitations such as, inferior image quality and lack of a unique CT-number-to-electron density calibration [18–21]. Furthermore, they have only a limited field of view (FOV) capacity, which may not enclose the structure set in its entirety. For image segmentation and dose calculation, the insufficient image quality has recently been overcome by generating synthetic CTs (sCTs) with an artificial intelligence (AI) approach [22].

The aim of ART is to minimize the discrepancies between high dose volumes and the actual clinical target volumes (CTV) and therefore, to further broaden the therapeutic window. In ART for patients with NSCLC, this can be realized with one of the following adaptation strategies: sparing the OARs while leaving the dose delivered to the PTV unaltered (isoeffective scenario) or escalating the dose to the PTV without increasing the ipsilateral lung-dose (isotoxic scenario) [16]. By minimizing OAR-doses, the risk of toxic events can be reduced [3, 9, 23]. This increases the probability that patients will be eligible for immunotherapy [7, 24]. Several analyses showed that increased OAR-doses are associated with worse OS [4, 5, 25]. Moreover, no correlation between effect and different tumor types or locations has been yet determined. Several studies concluded that in lung cancer higher fractional doses lead to higher TCP and OS [26–28]. This was, however, questioned by the results of the RTOG-0617 study which showed that patients treated with 60 Gy had better progression-free survival (PFS) and OS rates than patients treated with 74 Gy [5]. In the 74 Gy arm higher doses to the heart, lung, and esophagus were delivered compared to the 60 Gy arm [5, 25]. Higher lung- and heart-doses were associated with worsened OS [4, 5, 25, 29], which may explain the better outcome of the 60-Gy arm. Further analysis of the patient population indicated that dose-escalation may improve OS rates for patients with radioresistant genotypes [30].

This study aims to determine the differences between planned and delivered doses. Furthermore, the maximum dosimetric benefits of isotoxic and isoeffective daily online ART approaches with deep inspiration breath-hold technique (DIBH) for target immobilization shall be determined.

## Methods

### Patient population and treatment workflow

In this retrospective treatment planning study 13 patients with stages III/IV NSCLC, previously treated with cCRT were identified (Table 1). The primary tumors of two patients with distant metastases were included in the study, while the secondary lesions were not.

**Table 1:** Patient characteristics

Patient	Sex	Age	Tumor staging				Primary GTV (cm^3^)	Subtype	Tumor location	D_presc_ (Gy)/fx
			T	N	M	Stage				
P1	m	70	T4	N0	M1	IVA	81.7	SCC	RML	60/30
P2	m	60	T4	N3	M0	IIIC	50.3	SCC	LLL	60/30
P3	m	83	T3	N3	M0	IIIC	29.9	SCC	RUL	60/30
P4	m	59	T3	N1	M0	IIIA	15.4	AC	RLL	60/30
P5	f	55	T4	N3	M0	IIIC	163.8	AC	RUL	60/30
P6	m	80	T4	N3	M0	IIIC	83.2	SCC	LUL	58/29
P7	m	80	T4	N0	M0	IIIA	165.8	SCC	LUL	60/30
P8	m	73	T3	N3	M0	IIIC	32.5	SCC	RUL	60/30
P9	f	69	T4	N2	M0	IIIB	312.1	SCC	RUL	60/30
P10	m	50	T4	N2	M1	IVA	82.4	SCC	RUL	60/30
P11	m	65	T4	N2	M0	IIIB	231.2	SCC	LUL	60/30
P12	m	71	T3	N3	M0	IIIC	116.6	AC	LML	56/28
P13	f	65	T4	N3	M0	IIIC	83.9	AC	RUL	60/30
		67.7 ± 9.6 (49–80)					111.4 ± 83.0 (15.4–501.9)			

The patient, tumor and fractionation characteristics are presented, where f = female, m = male, GTV = gross target volume, SCC = squamous cell carcinoma, AD = adenocarcinoma, LLL = left lower lobe, RLL = right lower lobe, LML = left middle lobe, RML = right middle lobe, LUL = left upper lobe, RUL = right upper lobe, D_presc_ = prescription dose and fx = number of fractions. Tumors were staged according to the AJCC TNM staging system version 8. The average of the age and primary GTV size is displayed as mean ± standard deviation (ranges)

The mean treatment period was 47.5 days ± 4.0 days (range: 40–54 days). All patients were treated on a linear accelerator (VersaHD, Elekta AB, Stockholm, Sweden) with 10MV volumetric modulated arc therapy (VMAT). Treatment planning CTs (pCTs) and all dose deliveries were performed using a computer-controlled DIBH for target immobilization (ABC (Active Breathing Coordinator, Elekta AB, Sweden): 6, Catalyst (Catalyst, C-RAD, Sweden): 7). Therefore we did not consider an ITV but define GTV-PTV margins (axial: 10 mm, inferior-superior: 15 mm) [31]. For each patient, daily kV-based CBCT (XVI 5.0, Elekta AB, Sweden) scans were acquired for patient positioning. These scans were also obtained in multiple breath-hold-phases (“stop-and-go” breath-hold-only approach), immediately prior every treatment.

### Study image data preparation

The CBCT scans were reconstructed, rigidly registered to the pCTs using the clinically used registrations, in which a tumor match was favored and exported with a slice thickness of 3 mm to the treatment planning system (Monaco 5.11, Elekta). These CBCTs were converted into sCTs in a dedicated research software (ADMIRE, Elekta). For sCT generation this software utilizes a pre-trained artificial neural network [22]. Retrospectively, an expert physician contoured the GTV, heart, lungs, esophagus, spinal cord (sc), and the patient outline on the sCTs of the first fractions (sCT_1_) for all patients. These structures were deformably registered in an unsorted manner from the sCT_1_ of a patient to the remaining sCTs (sCT_2−n_) of the patient using deformable image registration (DIR). The deformed structures were retrospectively reviewed and corrected for all sCTs of every patient, if necessary, by the same expert physician. The same window and level settings were used for correcting all structures in all sCTs of the same patient, respectively. An additional structure ipsilateral lung minus GTV (lung_ipsilateral_) was created for all sCTs. According to the clinical protocol all PTV_2−n_ were generated out of the GTV_2−n_ with GTV-PTV margins of 10 mm in axial and 15 mm in inferior-superior directions. These margins were used unaltered throughout the study and are in agreement with the margins which were used in the RTOG-0617 study protocol [5].

### Treatment planning and dosimetric analysis

Initial VMAT treatment plans were generated in a Monte Carlo based treatment planning system (Monaco 5.11, Elekta) with a grid spacing of 3 mm and a statistical uncertainty of 1% per dose calculation. Dosimetric constraints for inverse treatment planning were: V_20Gy_(lung_ipsilateral_) ≤ 37%, V_20Gy_(lung_total_) ≤ 30%, ipsilateral mean lung dose (MLD_ipsilateral_) ≤ 20 Gy, V_5Gy_(heart) as low as possible, V_35Gy_(heart) < 10%, mean heart dose (MHD) < 10 Gy, D_0.1%_(sc) < 50.5 Gy and the mean esophagus dose (MED) < 34 Gy, while 95% of the PTV should be covered with the prescription dose (D_presc_). Except for the heart the dosimetric constraints were adopted from RTOG-0617 [5]. The tolerance doses of the heart were further reduced, because V_5Gy_(heart), V_35Gy_(heart), and MHD are potentially associated with cardiac events [23, 32, 33]. All treatment plans were clinically accepted for patient treatment and optimized for maximum dose conformality.

The initial treatment plan of each patient was recalculated with identical control point settings, grid size, statistical uncertainty, and an isocenter which resulted from the image registration process onto sCT_2−n_. Assuming only interfractional and neglecting residual intrafractional motion in DIBH [34] this scenario represents the daily delivered dose to the patient with IGRT (IGRT scenario). For the two adaptation approaches, new treatment plans were re-optimized on sCT_2−n_. Therefore, a new isocenter was set in the center-of-mass of the daily PTVs. For the isoeffective scenario the ipsilateral lung constraints of the initial prescription template were iteratively reduced as long as the target coverage remained adequate. Each treatment plan was normalized to cover 95% of the actual PTV_2−n_ with D_presc_. For the treatment plans of the isotoxic approach an equivalent procedure was applied with increasing the target dose. Subsequently, the V_20Gy,2−n_(lung_ipsilateral_) was normalized to the initial treatment plan V_20Gy,1_(lung_ipsilateral_) … with a maximum normalization between 90% and 110%. For three patients, the lungs were cropped due to a reduced FOV in the CBCTs. For these patients, the absolute initial ipsilateral lung volume was taken for normalization. In addition, a maximum dose for the D_95%_ (PTV) of 3.3 Gy per fraction was implemented in accordance with the 2.2–3.8 Gy of RTOG-1106 study protocol. Our lower maximal fractional dose of 3.3 Gy was chosen to preferably stay below the maximum total dose of 80.4 Gy of the RTOG-1106. Their study showed no adverse effects for this dose-escalation [35]. To avoid uncertainties due to a DIR-based dose accumulation, the resulting dose-volume histogram (DVH)-parameters of each fraction were evaluated independently. To enable using the standard DVH-parameters for cumulative dose distributions (like V_20Gy_) each fraction was evaluated with the total D_presc_. Thus, the fractional dose limit of 3.3 Gy (D_95%_(PTV)) corresponded to a limit of 100 Gy (D_95%_(PTV)) in the total D_presc_. In isotoxic cases where D_95%_(PTV) > 100 Gy was feasible, the treatment plan was normalized, so that 100 Gy covered 95% of the PTV. The mean value of all fractions of each patient was considered as an estimation of the dose accumulation.

The dose distributions of all fractions were evaluated regarding the GTV and PTV coverage, V_20Gy_(lung_ipsilateral_), MLD_ipsilateral_, MLD_contralateral_, MHD, V_5Gy_(heart), MED, and the D_0.1%_(sc). Furthermore, the equivalent dose in 2 Gy-fractions (EQD_2_) was determined using the linear quadratic model [36] with a specific tissue characterization ratio α/β = 8.2 Gy for the GTV and PTV [37]. The dosimetric parameters of the initial treatment plans were compared to the resulting plans of the IGRT, isoeffective, and isotoxic scenarios.

**Fig. 1:**
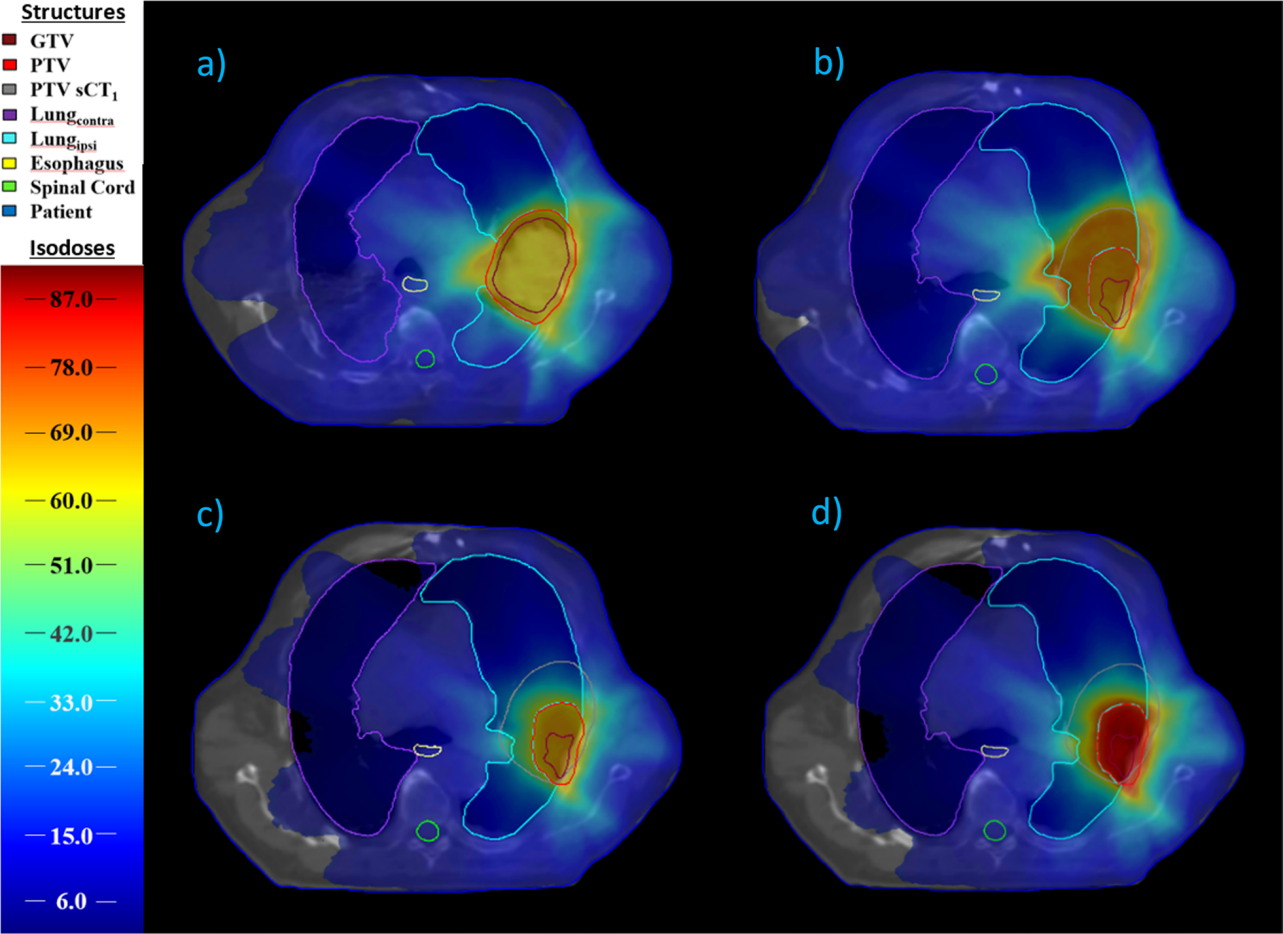
Exemplary dose distributions of a representative patient. Shown are dose distributions (**a**) of the original treatment plan, (**b**) of the last treatment fraction without adaptive radiotherapy (ART), (**c**) of an isoeffective ART, and (**d**) of an isotoxic ART treatment plan

In Fig. 1 the dose distributions in the axial isocenter plane of the initial treatment plan (Fig. 1a) on sCT_1_ of a representative patient is compared to the IGRT-, isoeffective- and isotoxic-dose distributions on sCT_30_. One can see the regressed GTV in sCT_30_ (Fig. 1b–d).

### Statistical analysis

The aforementioned dose-volume relationships for IGRT, isoeffective and isotoxic ART approaches were compared to the initial treatment plan results and tested for statistically significant (*p* < 0.05) differences using a non-parametric Wilcoxon signed-rank test in R (RStudio 1.4.1717, PBC, Boston, MA). Pearson’s correlation coefficients (PCC) were determined to analyze potential correlations between GTV-regression and analyzed DVH-parameters.

### Ethics statement

This investigation was performed according to the principles of the Declaration of Helsinki and after Institutional Review Board (IRB) approval (2018-836R-MA). All data was anonymized prior to inclusion.

## Results

### GTV-regression

**Fig. 2:**
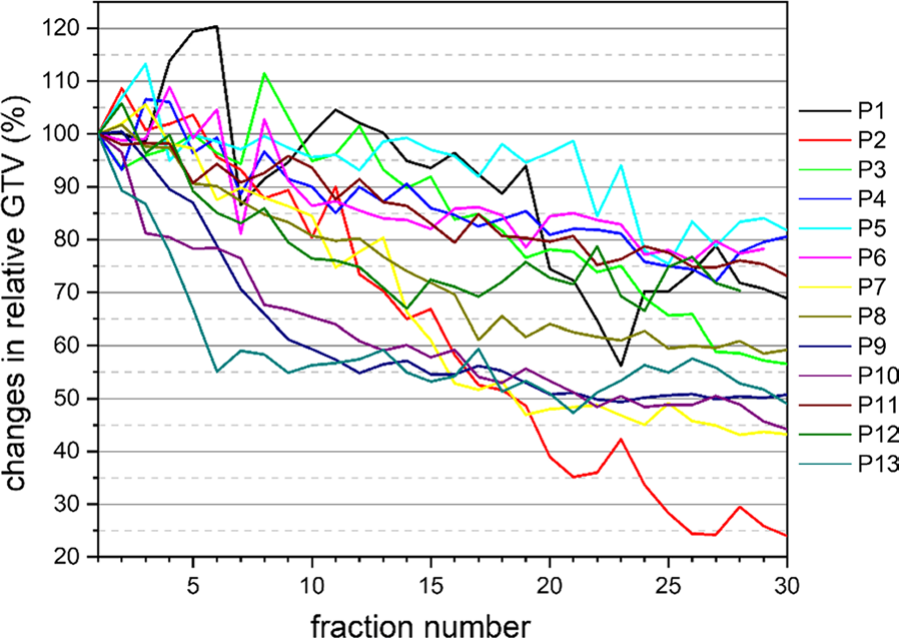
Daily volume changes of the gross target volume (GTV). Displayed are GTV dynamics for 13 patients (P1–P13) with locally advanced non-small cell lung cancer

The mean decrease in GTV on the final sCTs was 59.9% ± 15.8% (range: 24.0–81.8%) compared with the initial GTV, corresponding to a mean reduction of 1.4% ± 0.6% per treatment fraction and 0.9% ± 0.3% per day (Fig. 2).

### Dosimetric analysis

The median DVH-parameters of the initial treatment plans and the three scenarios are presented in Table 2.

**Table 2:** DVH-parameters

Structure	Metric	Median ± Interquartile range (min—max)			
		Original treatment plan	IGRT	Isoeffective ART	Isotoxic ART
GTV	D_95%_ (Gy)	60.5 ± 0.9 (59.0–62.3)	60.5 ± 2.0 (41.4–63.3)*	60.4 ± 1.6 (58.0–63.6)*	70.5 ± 9.4 (58.4–122.2)*
	Rel. D_95%_ (%)	100.0	100.2 ± 1.4 (68.4–103.9)*	99.9 ± 1.9 96.8–105.0)*	116.0 ± 13.5 (96.3–236.8)*
	EQD_2_ (D_95%_) (Gy)	60.6 ± 1.1 (58.8–62.8)	60.6 ± 2.4 (38.8–64.0)*	60.5 ± 1.9 (57.6–64.3)*	72.9 ± 11.9 (58.1–147.0)*
	Rel. EQD_2_ (D_95%_) (%)	100.0	100.3 ± 1.7 (64.1–104.7)*	99.9 ± 2.2 (96.1–106.0)*	119.6 ± 17.0 (95.4–234.0)*
PTV	D_95%_ (Gy)	60.0 ± 0.0 (56.0–60.0)	57.9 ± 5.8 (16.4–62.1)*	60.0 ± 0.0 (55.8–60.2)	65.5 ± 8.1 (53.5–100.0)*
	Rel. D_95%_ (%)	100.0	97.1 ± 7.3 (27.4–104.9)*	100.0 ± 0.1 (99.8–100.2)	109.2 ± 12.0 (94.2–221.3)*
	EQD_2_ (D_95%_) (Gy)	60.0 ± 0.1 (56.0–60.0)	58.7 ± 6.8 (14.1–62.5)*	60.0 ± 0.0 (55.8–60.2)	66.4 ± 9.8 (52.3–113.1)*
	Rel. EQD_2_ (D_95%_) (%)	100.0	98.8 ± 8.6 (23.5–105.9)*	100.0 ± 0.1 (99.8–100.3)	110.6 ± 14.8 (92.3–188.6)*
Lung_ipsilateral_	MLD (Gy)	14.2 ± 2.4 (7.8–16.9)	14.7 ± 3.0 (7.7–21.8)*	12.3 ± 3.4 (6.6–17.7)*	13.8 ± 2.5 (7.3–20.6)*
	Rel. MLD (%)	100.0	103.6 ± 12.7 (23.5–105.9)*	92.8 ± 10.6 (99.8–100.3)*	99.2 ± 3.4 (92.3–188.6)*
	V_20Gy_ (%)	30.2 ± 12.3 (13.0–36.9)	31.3 ± 10.0 (12.7–48.8)*	26.6 ± 14.6 (10.7–40.7)*	30.2 ± 12.4 (13.0–37.0)
	Rel. V_20Gy_ (%)	100.0	105.2 ± 14.7 (74.1–141.8)*	90.7 ± 14.4 (67.9–116.3)*	100.0 ± 0.0 (99.6–100.3)
Lung_contralateral_	MLD (Gy)	6.2 ± 4.4 (3.0–12.9)	6.7 ± 4.8 (2.7–13.7)*	6.0 ± 4.9 (2.2–12.9)*	6.5 ± 4.9 (2.6–13.6)*
	Rel. MLD (%)	100.0	105.1 ± 13.9 (88.1–150.1 *	96.7 ± 10.8 (71.6–124.3)*	102.7 ± 17.6 (73.7–146.8)*
Heart	MHD (Gy)	6.2 ± 5.0 (0.8–9.4)	6.3 ± 5.3 (0.8–15.0)*	5.8 ± 5.4 (0.55–13.0)*	6.2 ± 5.6 (0.8–14.0)*
	Rel. MHD (%)	100.0	100.0 ± 32.7 (53.6–257.2)*	90.4 ± 20.1 (49.0–164.8)*	100.0 ± 30.1 (46.4–196.1)*
	V_5Gy_ (%)	34.7 ± 24.4 (9.3–59.1)	34.8 ± 26.4 (6.8–100.0)*	30.0 ± 21.7 (4.4–100.0)*	30.8 ± 20.7 (4.5–100.0)*
	Rel. V_5Gy_ (%)	100.0	100.5 ± 23.6 (53.8–187.3)*	90.7 ± 19.3 (48.3–169.3)*	93.0 ± 29.7 (55.3–180.4)*
Esophagus	MED (Gy)	11.9 ± 9.4 (4.0–24.7)	12.5 ± 11.6 (2.9–27.5)*	11.0 ± 10.5 (2.9–25.7)*	11.1 ± 9.4 (2.7–25.1)*
	Rel. MED (%)	100.0	102.7 ± 16.1 (51.3–134.6)*	97.2 ± 17.6 (32.0–130.1)*	96.5 ± 22.3 (35.7–147.6)*
Spinal Cord	D_0.1%_ (Gy)	20.3 ± 6.1 (11.3–26.1)	21.3 ± 5.9 (10.6–37.6)*	20.4 ± 6.4 (8.8–29.8)*	21.2 ± 6.8 (7.8–31.0)*
	Rel. D_0.1%_ (%)	100.0	105.1 ± 11.1 (84.7–209.8)*	102.1 ± 15.3 (66.1–140.3)*	104.8 ± 16.1 (68.3–188.1)*

The absolute and relative median values of the DVH-parameters ± Interquartile ranges as well as (minimum-, maximum doses) for the gross target volume (GTV), planning target volume (PTV) and organs at risk are presented for the dose distributions of the following scenarios: original treatment plan (sCT_1_), after IGRT (sCT_1−n_), with isoeffective ART (sCT_1−n_), with isotoxic ART (sCT_1−n_). The abbreviations EQD_2_, MLD, MHD and MED are short for the equivalent dose in 2 Gy-fractions, mean lung dose, mean heart dose and mean esophagus dose, respectively. Statistically significant differences to the initial treatment plans are marked with an asterisk (*)

While the GTV-coverage remained intact in the IGRT scenario, the median D_95%_(PTV) was 1.6 Gy ± 4.2 Gy and the median EQD_2_(D_95%_(PTV)) 1.7 Gy ± 5.0 Gy lower than initially planned. The D_95%_(GTV) and D_95%_(PTV) were below 95% volume coverage in 21 (5.4%) and 108 (27.9%) fractions. Averaged over all treatment fractions of each patient the GTV and PTV coverage of 1 (7.7%) and 5 patients (38.5%) were too low, respectively. In the IGRT scenario all analyzed OAR-parameters were slightly higher than initially predicted. Compared to the initial treatment plan the median V_20Gy_(lung_ipsilateral_), MLD_ipsilateral_, V_5Gy_(heart) and MHD were 1.1% ± 4.4%, 0.5 Gy ± 1.8 Gy, 0.2% ± 6.7%, and 0.2 Gy ± 1.2 Gy higher. The V_20Gy_(lung_ipsilateral_), MLD_ipsilateral_, and the MHD were violating the previously mentioned constraints of 37%, 20 Gy, and 10 Gy in 69 (17.8%), 6 (1.6%), and 41 (10.6%) fractions and on average for 3 (23.1%), 0, and 1 patients (7.7%), while these tolerances were never exceeded in the initial treatment plans.

The PTV-coverage was restored in the isoeffective ART plans. The above-mentioned constraints for the D_95%_(GTV) and D_95%_(PTV) were not violated in any fraction in the isoeffective treatment plans. Simultaneously, the ipsilateral lung and heart were spared by 3.1% ± 3.6% (median V_20Gy_(lung_ipsilateral_)), 1.4 Gy ± 1.3 Gy (median MLD_ipsilateral_), 2.9% ± 6.4% (median V_5Gy_(heart)), and 0.5 Gy ± 1.4 Gy (median MHD) compared to the initial plan. The OAR-sparing of the isoeffective approach is even more pronounced compared to the IGRT scenario with 3.7% ± 6.9% (median V_20Gy_(lung_ipsilateral_)), 1.9 Gy ± 2.7 Gy (median MLD_ipsilateral_), 3.0% ± 5.7% (median V_5Gy_(heart)), and 0.6 Gy ± 1.3 Gy (median MHD). The constraints for the V_20Gy_(lung_ipsilateral_), MLD_ipsilateral_, and the MHD were exceeded for 7 (1.8%), 0, and 13 (3.4%) fractions and on average for no patient, respectively.

In the dose-escalation scenario median dose-escalations of 10.0 Gy ± 8.1 Gy (D_95%_(GTV)), 12.4 Gy ± 10.3 Gy (EQD_2_(D_95%_(GTV))), 6.6 Gy ± 8.9 Gy (D_95%_(PTV)), and 8.1 Gy ± 13.7 Gy (EQD_2_(D_95%_(PTV))) were achieved. The aforementioned constraints for the D_95%_(GTV) and D_95%_(PTV) were violated in 0 and 12 (3.1%) cases, respectively. The constraints for the V_20Gy_(lung_ipsilateral_), MLD_ipsilateral_, and the MHD were violated in 0, 7 (1.8%), and 27 (7.0%) fractions, respectively. In the accumulated dose no constraints were violated for any patients.

The IGRT scenario increased the median D_0.1%_(sc), MED, and the MLD_contralateral_ compared with the initial TPs. In the two ART approaches these median DVH-parameters were lower compared with the IGRT scenario. The previously defined constraints (D_0.1%_(sc) < 50.5 Gy, MED < 34 Gy, V_20Gy_(lung_total_) ≤ 30%) were not violated in any fraction of the three scenarios. The ipsilateral lung volume changes resulted in higher MLD_ipsilateral_-doses in some fractions. That explains the higher maximal MLD_ipsilateral_-doses in the IGRT, isoeffective, and isotoxic scenarios. Higher maximum doses for the V_5Gy_(heart) and MHD in the three scenarios were caused by GTV movements towards the heart in 2 patients.

### DVH-parameter correlation with GTV-regression

A PCC between the GTV-volume and a DVH-parameter ≥ ± 0.5, which indicates an at least moderately strong correlation, was found for the V_20Gy_(lung_ipsilateral_) = −0.6 in the IGRT scenario. This indicates that a decreasing GTV-volume leads to an increase in ipsilateral lung-dose with IGRT-only. For the isoeffective scenario, on the other hand, the PCC of V_20Gy_(lung_ipsilateral_) was + 0.6 which implies a decrease of ipsilateral lung-dose with a decrease in GTV-volume. In the isotoxic scenario with a PCC of the D_95%_(GTV) = −0.5, an increasing GTV-dose with decreasing GTV-volume was observed.

### Fraction-wise analysis of a representative patient

**Fig. 3:**
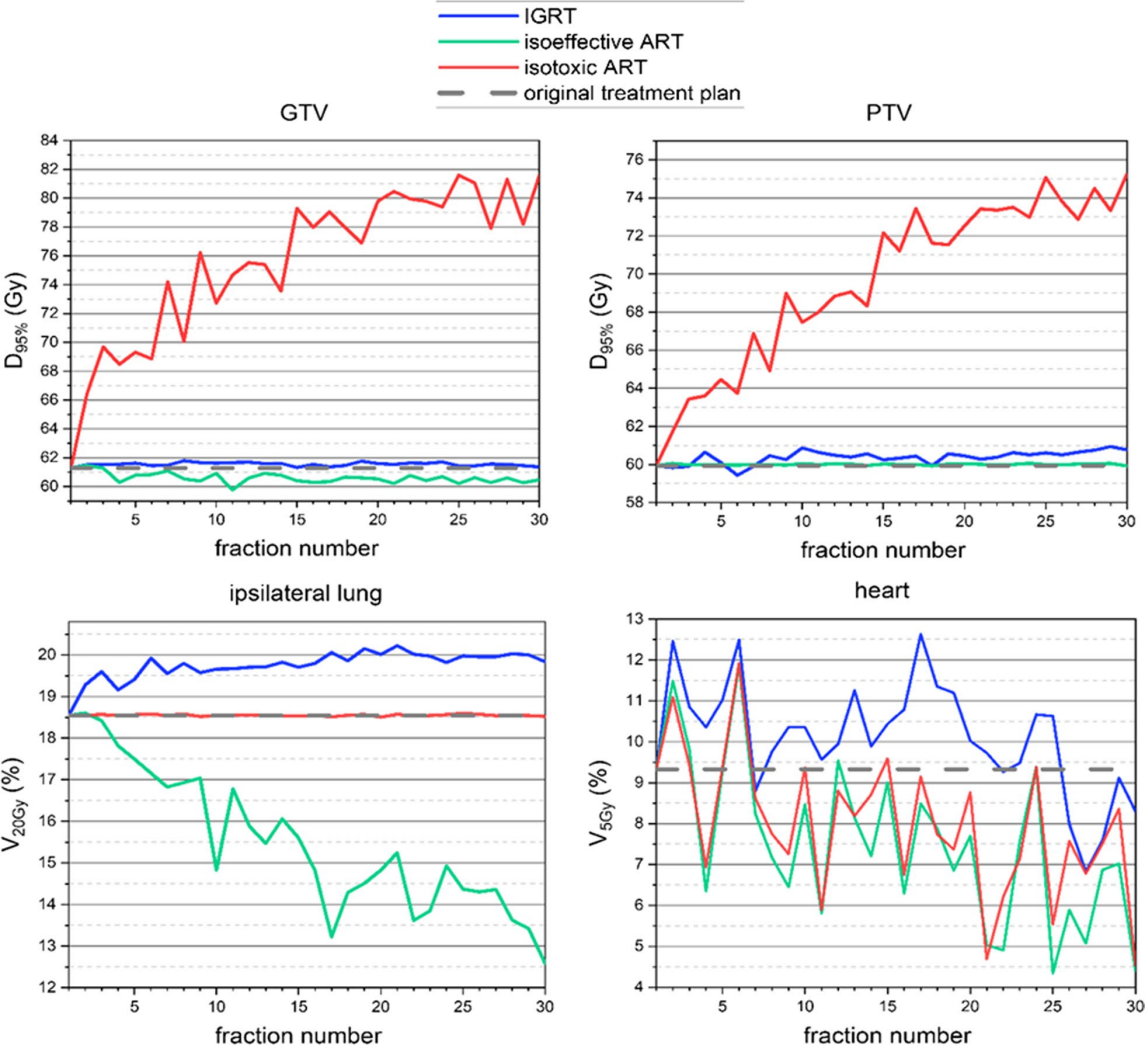
Fraction-wise analysis of dose-volume histogram (DVH)-parameters of a representative patient. The DVH-parameters of the gross target volume (GTV) (D_95%_(GTV)), planning target volume (PTV) (D_95%_(PTV)), ipsilateral lung (V_20Gy_(lung_ipsilateral_)) and the heart (V_5Gy_ (heart)) are shown as a function of treatment fractions for the three scenarios - without adaptive radiotherapy (ART) (blue), isoeffective ART (green), and isotoxic ART (red). The dashed grey line represents the respective DVH-parameter of the initial treatment plan

Fraction-wise dosimetric analysis of the D_95%_(GTV), D_95%_(PTV), V_20Gy_(lung_ipsilateral_), and V_5Gy_(heart) over all treatment fractions of one representative patient (with median GTV-reduction) with D_presc_ = 60 Gy is presented in Fig. 3. In the last sCT of this patient, the GTV-volume was reduced to 59.3% of the initial GTV. The mean D_95%_(PTV) was 69.8 Gy in the isotoxic scenario, whereas in the isoeffective scenario the mean V_20Gy_(lung_ipsilateral_) was reduced by 3.0%. One can see the aforementioned dose-escalation (D_95%_(GTV) and D_95%_(PTV)) and the lung-dose sparing (V_20Gy_) in the isotoxic and isoeffective scenario, respectively. For the V_5%_(heart) a strong fluctuation is noticeable in every scenario. In the IGRT scenario an increase in V_20Gy_(lung_ipsilateral_) by 1.6% was found while the target coverage remained intact over all fractions.

## Discussion

To our knowledge, this is the first study evaluating the dosimetric benefits of daily isotoxic and isoeffective ART approaches for patients with stage III NSCLC on daily CBCT-based sCTs. The generated sCTs have the advantage of preserving the daily anatomy and providing accurate electron density allocations, comparable to those of the pCTs. While dosimetric errors were reported to be typically less than 1% with this method [22], the errors were further minimized by comparing dosimetric results to treatment plans optimized on the sCT_1_ and not pCTs.

Dose accumulation is mostly based on deformation vector fields (DVFs) generated with a DIR. The accuracy of this method depends strongly on the image resolution, artifacts, and image distortion [38, 39]. Furthermore, the generation of the DVFs is a problem with no unique solution due to too many degrees of freedom [40]. This eventually results in dose inaccuracy in DIR-based dose accumulation [39]. This inaccuracy becomes more pronounced for regressing structures because their mass is not preserved [41, 42]. That makes dose accumulations challenging when shrinking tumors are included [41, 42]. Zhong et al. reported a reduced MLD by around 2 Gy for isoeffective ART of patients with NSCLC from 17.3 Gy to either 15.2 Gy, 14.5 Gy or 14.8 Gy when using three different DIR algorithms for the same data. That yields an algorithm-based MLD fluctuation of 0.7 Gy (4%) between the three DIR algorithms [42].

Without adaptation, the GTV-coverage remained adequate, but the median D_95%_(PTV) decreased by 1.6 Gy ± 4.2 Gy. In a study by Luo et al. in which the treatment plans of 24 NSCLC patients were retrospectively recalculated on weekly CBCTs, only 81.5% of the PTVs received the D_presc_ instead of 95.6% of the initial plans [43]. The evaluated OAR-doses were exceeded for all analyzed DVH-parameters. Since the known OAR tolerances are today solely based on the initial treatment plans [44], OAR tolerances are likely to change for ART. The analysis of Hoegen et al. yielded a lung dose increase (MLD, V_20Gy_) for 10 patients with NSCLC by daily recalculation on CBCTs without plan adaptations comparable to the results of this work [45].

### Benefits of isoeffective ART

In isoeffective ART, treatment plan adaptations to the regressed tumor volume allowed a restoration of the PTV coverage while minimizing the OAR-doses. The median MLD_ipsilateral_ and V_20Gy_ (lung_ipsilateral_) were 1.4 Gy ± 1.3 Gy and 3.1% ± 3.6% lower than initially planned. In the study of Hoegen et al. with weekly isoeffective plan adaptations for 10 patients with NSCLC, this effect is less evident, which can be explained by less frequent adaptations [45]. Guckenberger et al. obtained weekly CT for 13 patients with NSCLC to simulate ART. At week 3 or 5, or both, the treatment plans were adjusted. The results of their isoeffective ART at weeks 3 and 5 were comparable to our results for MLD. The effect that they achieved similar results with only 2 adaptations could be due to larger mean GTV-volumes and higher GTV-regression rates in their patient collective [46].

In a recent retrospective study, an isoeffective ART group had a 4.3 Gy lower median MLD and 3.6 Gy lower median MHD than a non-ART group. It is of notice that this group in contrast to our study used smaller margin sizes for the ART group, which explains the large MLD and MHD sparing [24]. Despite their retrospective nature, these results demonstrate the possibilities of isoeffective ART. Only 20%, 7%, and 0.4% of the patients in the ART group experienced pneumonitis with a grade ≥ 2, ≥ 3, or lethal instead of 50%, 21%, and 6% of the patient population without ART. The OS and PFS after two years increased by 13% and 8% in the ART group compared to the non-ART control group, respectively [24]. In this study, new 4D-CTs for each adaptation were required. If CBCTs, which are already used for patient positioning, or CBCT-based sCTs could be used for the treatment plan adaptation, the extra dose of the 4D-CTs could be avoided.

In some treatment fractions the OAR-constraints were violated due to tumor movement towards the heart and lung in two patients. Furthermore, lung volume changes caused higher V_20Gy_ of the ipsilateral lung in some fractions. To gain the initial coverage, the OAR doses were exceeded in the isoeffective ART scenario.

### Benefits of isotoxic ART

It is still a matter of discussion which patients could benefit from an isotoxic ART approach with a target dose-escalation and which upper dose limits are appropriate. Improved outcomes in dose-escalation studies for patients who received RT alone or after inducting chemotherapy up to 84 Gy [47] and 103 Gy [26] were found. Ramroth et al. showed a prolonged median survival with dose-escalation for patients without chemotherapy in a meta-analysis involving 3795 patients from 25 trials. The time-corrected EQD_2_ ranged from 36.4 Gy to 80.8 Gy. With cCRT the opposite occurred which might be due to higher levels of toxicity [48]. In the study trial RTOG-0617 patients with cCRT who were treated with a D_presc_ of 60 Gy had better OS rates than patients who were treated with 74 Gy [5]. Schild et al. showed the more frequent occurrence of adverse events grade III or greater with dose-escalation in a meta-analysis involving 3600 patients from 16 trials [49]. Different to the RTOG-0617 and the study of Schild et al. for the isotoxic ART scenario, a fractional dose-escalation was chosen in this work to avoid tumor repopulation due to treatment prolongation [11, 50, 51] and the OAR-doses should not exceed the initially planned values to avoid the increased occurrence of adverse events.

The median D_95%_(GTV) was 10.0 Gy ± 8.1 Gy higher compared to the initial plans. That is a larger dose increase than the result of the isotoxic ART in the prior mentioned study by Guckenberger et al. with a mean escalation of the D_95%_(GTV) by 5.7 Gy. Remarkably, this dose-escalation was achieved by adapting only twice in the treatment period [46]. The median EQD_2_(D_95%_(PTV)) was 8.1 Gy ± 13.7 Gy higher than the initial treatment plans. That is lower than the mean EQD_2_(D_95%_(PTV)) dose-escalation of 13.4 Gy which was obtained by Weiss et al. by adapting treatment plans of 10 patients with NSCLC in weeks 2 and 4. That they achieved a higher dose-escalation with only 2 adaptations might be explainable by our dosimetric limit of the D_95%_(PTV), the larger GTV-reduction they reported, the chosen α/β-ratio, and furthermore, they escalated the dose until the MLD exceeded the initial dose by 1 Gy [52]. The ongoing RTOG-1106 study compares normofractionated patients with patients for whom a mid-treatment PET-CT was used for individualized dose-escalation. The median target dose with one adaptation was 71 Gy. The 2-year in-field local–regional control and primary tumor control of the patients in the adaptation arm was 10.7% and 17.1% higher than for the normofractionated patients [35]. An adaptation based on a PET-CT to the residual active volume might lead to more promising results, but is time-consuming and leads to additional doses which cannot be neglected and therefore, are unsuitable for daily adaptations [45, 53].

The normalization of the isotoxic ART treatment plans to the V_20Gy_ of the ipsilateral lung could lead to an excess of the remaining DVH parameters in cases where the V_20Gy_ was not the limiting OAR-constraint. Furthermore, we allowed dose normalization ratios between 90% and 110%, which could also cause a violation of OAR constraints in the isotoxic ART scenario.

### Limitations and benefits of the proposed approach

Having one physician performing all image segmentation reduces possible bias introduced by inter-individual assessments. Furthermore, contouring retrospectively in an unsorted sCT-order minimized a potential bias in favour of any method. Nevertheless, the volume changes of the GTV seen in Fig. 2 cannot be solely attributed to daily changes in tumor size, but could also be influenced by the resolution of the sCT and intraobserver variability of the structure delineation, which, however, is already covered by the GTV-PTV margin. Furthermore, accurate image segmentation of the heart was expected to be challenging since it is mostly located at the periphery of CBCT scans, where soft tissue discrimination is weak. Therefore, dosimetric consequences in this structure will be more likely prone to errors. Daily fluctuations of certain DVH-parameters as displayed in Fig. 3 demonstrate the difficulty of a reproducible treatment scenario and subsequent image segmentation. Especially daily fluctuations in the structure which is used for dose normalization (PTV or ipsilateral lung) will lead to dosimetric deviations. This is of particular interest when adaptations are performed on a regular but not daily basis, e.g. once weekly [28].

The relatively small FOV of conventional CBCT scanners resulted in only partially visible lungs for three patients. A larger FOV would be advantageous but might imply higher imaging doses.

Although daily ART becomes feasible with modern linear accelerators it is typically still time-consuming. The benefit of ART decreases from midterm to weekly and daily ART [28]. Woodford et al. stated that a tumor regression of at least 30% after 20 treatment fractions is worth replanning. A predictor of the time points for every patient, where the replanning is worth the effort or a predictor for OAR toxicities would be beneficial. This however, implies that pre-treatment volume images are available with a sufficiently high image quality.

Changes in the fractional dose of the isotoxic treatment plans could affect the patient’s anatomy differently than the normofractionated ones. Higher fractional doses could further increase the effect of the GTV-regression and therefore, as well of the dose-escalation [42]; nonetheless, fractionation-related effects remain yet uncertain. In addition, the tested ART strategies are pre-treatment adaptations, which address only interfractional and not intrafractional anatomic changes, thus the effect is larger than that of offline ART strategies, but could underestimate the effect of real-time online ART Strategies.

Our approach for two different treatment strategies namely, isoeffective ART and isotoxic ART was feasible, confirmed by an accurate dosimetric analysis of daily ART. All treatment plans were calculated for the same patient collective which makes them reproducible. In the isoeffective scenario the OAR-doses were spared with adequate target-coverage in all treatment fractions. In the isotoxic treatment plans the dose to the target volume could be escalated without relevantly increasing the OAR exposure.

### Potential future implementations

sCT-guided ART can be implemented on conventional linear accelerators that are equipped with a CBCT scanner. However, a separate CBCT to sCT conversion algorithm might need to be trained for different CBCT scanners [22]. Alternatively, some of the new linear accelerators, such as the Ethos (Varian Medical Systems, Palo Alto, CA), are already equipped for online ART [54].

Other possibilities are adaptations based on PET-CT or CT, which, however, are associated with additional radiation exposure [45, 53], or MR-guided adaptations. The advantages of adapted MR-guided radiotherapy are that MR imaging is possible without ionizing radiation [55, 56], MR images have a better soft tissue contrast than in CT, which facilitates delineation of the OAR and the target [57–60], and MR-guided ART allows for real-time monitoring [58, 61] and can be combined with functional imaging to assess tumor response to therapy. This offers the possibility to adjust the therapy based on the biological information [58]. The disadvantages of MR-guided ART are the low availability of MR linacs [62], the required manpower that has to be present during the entire treatment [60], and the treatment duration [61–63].

## Conclusion

Lung-IGRT results in appropriate interfractional target-coverage, at expenses of higher OAR exposure. Both online issoeffective and isotoxic ART achieve adequate OAR-sparing dosimetry, whereas the latter allows dose-escalated PTV coverage. Ongoing research will elucidate the clinical applicability of this approach.

## Data Availability

The datasets used and analysed during the current study are available from the corresponding author on reasonable request.
